# Real‐world treatment outcomes of sofosbuvir‐based regimens for treatment of chronic hepatitis C with and without human immunodeficiency virus co‐infection

**DOI:** 10.1002/jgh3.12869

**Published:** 2023-01-26

**Authors:** Suparat Khemnark, Weerawat Manosuthi

**Affiliations:** ^1^ Department of Medicine Bamrasnaradura Infectious Disease Institute, Ministry of Public Health Nonthaburi Thailand

**Keywords:** human immunodeficiency virus/hepatitis C virus co‐infection, real‐world setting, sofosbuvir‐based regimens, treatment for chronic hepatitis C

## Abstract

**Background and Aim:**

The efficacy of sofosbuvir (SOF)‐based regimens in the treatment of chronic hepatitis C (HCV) patients with and without human immunodeficiency virus (HIV) co‐infected patients in real‐world setting is limited.

**Methods:**

This was a retrospective cohort study, conducted between 1 January 2017 and 31 December 2021 at Bamrasnaradura Infectious Disease Institute, Thailand. All HCV patients received 12 weeks of SOF‐based regimens and had follow‐up for at least 12 weeks after therapy discontinuation. The primary outcome was sustained virological response (SVR) at 12 weeks after the end of treatment. Treatment outcomes were compared between HCV patients with and without HIV co‐infection.

**Results:**

A total of 163 patients were included in the study, 130 (79.8%) were HCV/HIV co‐infected, and 33 (20.2%) were HCV mono‐infected. Of all, 106 (64%) patients received SOF and ledipasvir. Genotype 1 (GT1) was predominant at 66.4%, followed by GT3 at 22.2%, and GT6 at 11.4%. Overall SVR was 96.9%. SVR in HCV mono‐infected was 96.9% and SVR in HIV‐HCV co‐infected patients was 96.9%. The factor associated with SVR was HCV genotype (*P* = 0.001). Patients with HCV GT6 had lower SVR rates compared with GT1 and GT3 patients (83.3%, 100%, and 97.1% [*P* = 0.000] respectively). There was no association between SVR and other factors such as gender, age, BMI, underlying cirrhosis, baseline HCV viral load, or prior treatment history (all *P* > 0.05). All patients completed 12‐week SOF‐based treatment.

**Conclusion:**

In real‐world setting, HCV treatment with SOF‐based regimens between patients with and without HIV co‐infection showed high rates of SVR. SOF‐based regimens were highly efficacious and tolerated.

## Graphical abstract

In real‐world setting, hepatitis C virus treatment with sofosbuvir (SOF)‐based regimens between patient with and without human immunodeficiency virus co‐infection showed similar high rates of sustained virological response. SOF‐based regimens were highly efficacious and tolerated.

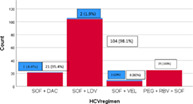

## Introduction

It is estimated that there are 71 million people who are chronically infected with the hepatitis C virus (HCV) globally, with a prevalence of 1.1%.^1^ In Thailand, the overall prevalence is predicted to be approximately 1–2%.^2^ Nonetheless, this prevalence is up to 6.9% in some provinces.^3^ The prevalence of HCV co‐infection with human immunodeficiency virus (HIV) in Thailand is 4.1%^4^ and as high as 77.6% in patients with HIV and history of intravenous drug usage.^5^ HCV infection in HIV‐positive patients leads to aggressive liver diseases with advanced fibrosis and earlier progression to end‐stage liver diseases.^6^, ^7^, ^8^ The main objective of HCV therapy is to achieve the eradication of the virus, defined as sustained virological response (SVR), which associates with reduced incidence of liver inflammation and fibrosis, hepatic decompensation, and hepatocellular carcinoma (HCC).^9^ In addition, SVR leads to decreased overall mortality from both hepatic and non‐hepatic causes.^10^, ^11^

Recent introduction of direct‐acting antivirals (DAAs) therapy has revolutionized the treatment landscape of chronic HCV. The treatment is effective with greater than 90% cure rate reported, well tolerated, and convenient given relatively short treatment duration.^12^, ^13^, ^14^, ^15^ DAAs have been available in Thailand since 2017. Since 2018, SOF and ledipasvir (LDV) in the form of fixed‐dose combination pill have been included in Thailand's National List of Essential Medicine and are assessable with free of charge for patients infected by HCV‐GT1, GT2, GT4, and GT6 with significant stage liver disease, in accordance with the national health policy for treating chronic hepatitis C. Nevertheless, SOF plus pegylated interferon (PEG‐IFN) plus ribavirin (RBV) combination therapy for 12 weeks remains the standard of care for patients infected with GT3, who are interferon supersensitive, regardless of underlying liver cirrhosis. Recently, the Thai government provided SOF/velpatasvir (VEL) that can treat pangenotypic chronic hepatitis C infection but only HCV patients with significant stage of liver disease are eligible to receive medication from the Thai government.

The aim of this study was to evaluate the effectiveness and safety of SOF‐based regimens for HCV‐infected patients with and without HIV infection.

## Methods

### Study design

This is a retrospective cohort study conducted at Bamrasnaradura Infectious Disease Institute, Nonthaburi, Thailand. We analyzed data consecutively from the medical records of all the chronic hepatitis C patients treated with SOF‐based regimens in the ambulatory clinic between 1 January 2017 and 31 December 2021. Cirrhosis was defined by fibroscan ≥12.8 kPa or APRI score >1.5 or from sonographic findings. The study was carried out in accordance with the ethical principles of the Declaration of Helsinki and was approved by the ethics committee of Bamrasnaradura Infectious Disease Institute (S019h/64_ExPD).

### Statistical analysis

Demographic data and baseline characteristics were analyzed using descriptive statistics. The mean ± SD was used when the numerical data were normally distributed while the median (interquartile range 25th and 75th, IQR) was used when skewed and frequencies (%) are used to describe characteristics in each group. Patients were classified into two groups as follows: (i) patients with HCV monoinfection and (ii) patients with HIV/HCV co‐infection. The categorical variables were compared using Chi‐square or Fisher exact test as appropriate. Continuous variables were compared using independent *t*‐test or Mann–Whitney *U* test. SVR12 were demonstrated in percentages (%). All statistical examinations were two‐tailed and *P*‐values less than 0.05 were considered statistically significant. Univariate was used to assess factors related to SVR12. *P*‐values less than 0.05 were considered significant. Multivariable logistic regression was performed only in variables with a *P*‐value <0.05 in univariate analysis. Statistical analysis was performed with the SPSS statistics software package version 26.0 (IBM Corp., Armonk, NY, USA).

## Results

A total of 163 patients were included. Of all, 130 patients were HIV/HCV co‐infected and 33 patients were HCV monoinfected. The characteristics of chronic hepatitis C patients were described and compared between the two study groups, as shown in Table 1. GT1 was predominant at 66.4%, followed by GT3 at 22.2%, and GT6 at 11.4%. The monoinfection and co‐infection groups were also comparable in terms of initial HCV viral load (1 120 000 [3270–15 900 000] *vs* 2 220 000 [280–33 900 000]) IU/mL and categorized in high baseline viral load (HCV RNA >6 000 000 IU/mL) (26.3% *vs* 24.3%). A total of 19 patients (11.7%) were treatment‐experienced. The prevalence of cirrhosis was not different between both groups (48.5% *vs* 44.4% in HCV monoinfection and HIV/HCV co‐infection respectively). All patients received at least 12 weeks of treatment, with one of the combination regimens in standard doses for chronic HCV infection.

**Table 1:** Baseline characteristics of all 163 patients

	Total	HIV status		*P*‐value
		HIV negative (33)	HIV positive (130)	
Age (years), mean (SD)	47.6 (10.9)	56.9 (12.5)	45.2 (9.1)	<0.001
Male, gender (%)	137 (84)	20 (60.6)	117 (90)	<0.001
BMI (kg/m^2^), mean (SD)	23.7 (4.5)	25.4 (5.4)	23.3 (4.2)	0.017
HCV genotype				0.003
1, *n* (%)	105 (66.4)	15 (50)	90 (70.3)	
3, *n* (%)	35 (22.2)	7 (23.3)	28 (21.9)	
6, *n* (%)	18 (11.4)	8 (26.7)	10 (7.8)	
HCV RNA (IU/mL), median (IQR)	2 175 000	1 120 000 (4 994 695)	2 220 000 (5 856 500)	0.853
<6 000 000 IU/mL, *n* (%)	42 (25.9)	8 (24.2)	34 (26.3)	
>6 000 000 IU/mL, *n* (%)	120 (74.1)	25 (75.8)	95 (73.7)	
Initial CD4 count, cells/mm^3^, mean (SD)	—	—	572 (271)	
Initial HIV VL, copies/mL				
<50			125	
>50			3	
ARV regimens				
TDF + FTC/3TC + EFV			55 (42.6)	
TDF + FTC/3TC + NVP			28 (21.7)	
TDF + FTC/3TC + LPV/r			8 (6.2)	
Other			38 (19.5)	
Prior treatment history				0.004
Treatment naïve, *n* (%)	144	24 (72.7)	120 (92.3)	
Treatment experience, *n* (%)	19	9 (27.3)	10 (7.7)	
Cirrhosis				0.450
No	88	17 (51.5)	71 (54.6)	
Yes	75	16 (48.5)	59 (45.4)	
APRI score				0.807
<0.5, *n* (%)	38 (23.8)	8 (24.2)	30 (23.6)	
0.5–1.5, *n* (%)	73 (45.6)	14 (42.4)	59 (46.5)	
>1.5, *n* (%)	49 (30.6)	11 (33.4)	38 (29.9)	
Other comorbidities				
Diabetes	19	7 (21.2)	12 (9.2)	0.055
Hypertension	28	12 (36.3)	16 (12.3)	0.001
Biochemical markers				
TB (mg/dL), median (IQR)	0.58 (0.50)	0.76 (0.59)	0.53 (0.42)	0.111
AST (U/L), median (IQR)	61 (61.5)	58 (67)	63 (54.8)	0.824
ALT (U/L), median (IQR)	65 (72.5)	47 (58)	67 (73.3)	0.176
ALP (U/L), median (IQR)	98 (45)	81 (32)	101 (44)	0.021
Albumin (g/dL), median (IQR)	4.3 (0.5)	4.2 (0.6)	4.3 (0.5)	0.109
INR, mean (SD)	1.00 (0.12)	1.13 (0.12)	0.98 (0.11)	0.002
WBC (/mm^3^), mean (SD)	6330 (1915.6)	6490 (1674.7)	6289 (1975.8)	0.591
Hemoglobin (g/dL), mean (SD)	14.22 (1.69)	13.56 (1.86)	14.39 (1.60)	0.011
Platelet (×10/L), mean (SD)	204 214 (74 991)	179 727 (66 931)	210 430 (75 886)	0.035
Creatinine (mg/dL), mean (SD)	0.93 (0.20)	0.88 (0.18)	0.94 (0.20)	0.118

Data are presented as mean (SD), median (IQR) or number (%).

3TC, lamivudine; ALP, alkaline phosphatase; ALT, alanine aminotransferase; APRI, aspartate aminotransferase‐to‐platelet ratio index; ARV, antiretroviral drugs; AST, aspartate aminotransferase; BMI, body mass index; EFV, efavirence; FTC, emtricitabine; HCV, hepatitis C virus; HIV, human immunodeficiency virus; INR, international normalized ratio; LPV/r, lopinavir/ritonavir; NVP, nevirapine; TB, total bilirubin; TDF, tenofovir; WBC, white blood cell.

**Figure 1:**
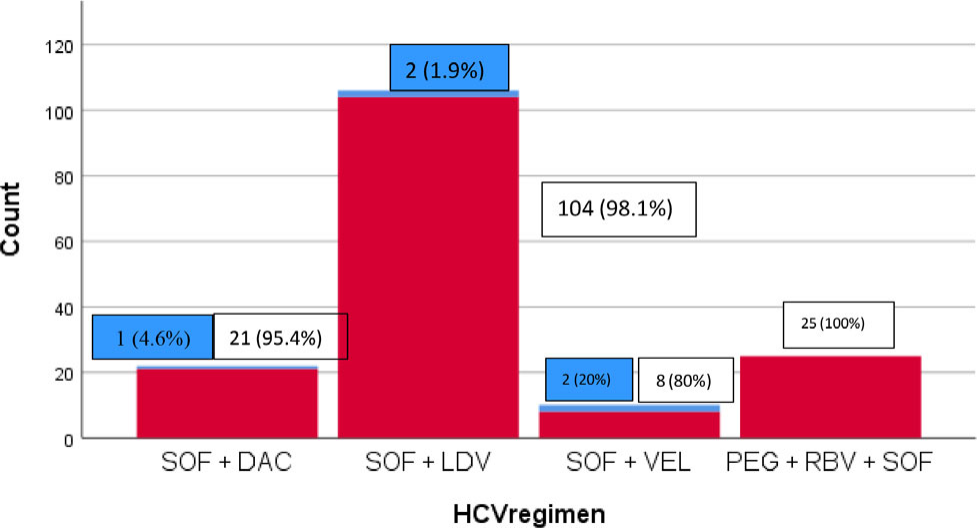
Hepatitis C virus treatment regimens. SVR (Yes, No): 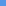, No; 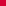, yes.

The details of treatment regimens are shown in Figure 1. Sixty‐five percent of the patients were treated with SOF/LDV with or without RBV, 15.3% (*n* = 25) with PEG‐IFN plus RBV plus SOF, 13.5% with DAC plus SOF with or without RBV, other with SOF/VEL (6.1%, *n* = 10). After 12 weeks, the overall SVR for the whole cohort was 96.9%. The response rate was not statistically different between the HCV mono‐infection group and without HIV/HCV co‐infection group (96.9% *vs* 96.9%; *P*‐value = 0.989). The univariate analysis determined the factors associated with the SVR was also performed on variables with significant findings (with *P*‐value <0.05 in univariate analysis; Table 2). SOF/VEL regimen showed lower rate of SVR compared with other regimens. Five patients (3.1%) did not achieve SVR and the baseline characteristics are demonstrated in Table 3. Univariate analysis showed lower SVR rate in patients with GT6 infection compared with patients with GT1 and GT3 infection (SVR rates 83.3%, 100%, and 97.14% respectively, *P* < 0.05).

**Table 2:** Factor associated with sustained virological response (SVR) by univariate analysis

	Total (163)	Achieved SVR 12	Not achieved SVR 12	Univariate (*P*‐value)
HIV status				0.733
HIV group	130	126 (96.9)	4 (3.1)	
Non‐HIV group	33	32 (96.9)	1 (3.1)	
CD4 count, cell/mm^3^	572 (271)	573 (274)	566 (224)	0.969
Gender				0.180
Male	137	134 (97.8)	3 (2.2)	
Female	26	24 (92.3)	2 (7.7)	
Age (years), mean (SD)	47.6 (10.9)	47.5 (10.87)	51 (13.21)	0.250
Age group				0.344
<65	150	146 (97.3)	4 (2.7)	
≥65	13	12 (92.3)	1 (7.7)	
BMI, kg/m^2^	23.58 (4.53)	23.67 (4.60)	23.89 (1.83)	0.273
BMI (group)				0.852
<25	113	109 (96.4)	4 (3.6)	
≥25	47	46 (97.9)	1 (2.1)	
Genotype				0.000
GT1	105	105 (100)	0	
GT3	35	34 (97.1)	1 (2.9)	
GT6	18	15 (83.3)	3 (16.7)	
HCV RNA (IU/mL)				0.753
Low (<6 000 000)	120	117 (97.5)	3 (2.5)	
High (≥6 000 000)	42	40 (95.2)	2 (4.8)	
HCV RNA (log10), mean (SD)	6.11	6.09 (0.88)	6.64 (0.50)	0.165
HCV treatment regimens				0.011
SOF + DAC	22	21 (95.4)	1 (4.6)	
SOF + LDV	106	104 (98.1)	2 (1.9)	
SOF + VEL	10	8 (80)	2 (20)	
PEG‐IFN + RBV + SOF	25	25 (100)	0 (0)	
Previous treatment				0.534
Yes	19	19 (100)	0	
No	139	134 (96.4)	5 (3.6)	
Cirrhosis				0.375
Yes	75	74 (98.7)	1 (1.3)	
No	88	84 (95.5)	4 (4.5)	
TB (mg/dL), median (IQR)	0.58 (0.50)	0.54 (0.50)	1.13 (0.93)	0.056
AST (U/L), median (IQR)	61 (61.5)	61 (61.5)	67 (82.3)	0.735
ALT (U/L), median (IQR)	65 (72.5)	65 (75)	79.5 (61)	0.716
ALP (U/L), median (IQR)	98 (45)	98 (44)	83.5 (39)	0.166
INR, mean (SD)	1.00 (0.12)	1.01 (0.13)	0.98 (0.08)	0.735
Hemoglobin (g/dL), mean (SD)	14.22 (1.69)	14.19 (1.70)	15.26 (0.67)	0.162
Platelet (×10^9^)	204 214 (74991)	203 702 (75420)	220 400 (64492)	0.625
Albumin (g/dL), median (IQR)	4.3 (0.5)	4.3 (0.6)	4.2 (0.8)	0.164
Albumin/globulin ratio, mean (SD)	1.16(0.26)	1.16(0.26)	1.11(0.26)	0.701
APRI score				0.913
< 0.5	37	36 (97.3)	1 (2.7)	
0.5–1.5	74	71 (95.9)	3 (4.1)	
>1.5	49	48 (97.8)	1 (2.2)	

Data are presented as mean (SD), median (IQR) or number (%).

ALP, alkaline phosphatase; ALT, alanine aminotransferase; APRI, aspartate aminotransferase‐to‐platelet ratio index; AST, aspartate aminotransferase; BMI, body mass index; CD4, cluster of differentiation 4; DAC, daclatasvir; GT, genotype; HCV, hepatitis C virus; HIV, human immunodeficiency virus; INR, international normalized ratio; LDV, ledipasvir; PEG‐IFN, pegylated interferon; RBV, ribavirin; SOF, sofosbuvir; SVR12, sustained virological response at 12 weeks after treatment; TB, total bilirubin; VEL, velpatasvir.

**Table 3:** Characteristics of patients who failed to response to treatment

No.	Regimen	Duration	Age	sex	HIV status, risk factor	ART	DM	HCV VL	GT	TN/TE	Cirrhosis
1	SOF/LDV	12	55	M	Yes (CD4 450 [20%], VL <20), Hx IVDU	Kivexa + NVP	No	1 464 901	6	TN	No
2	SOF + DAC	12	72	F	No	—	Yes	1 900 000	6	TN	No
3	SOF/LDV	12	47	M	Yes (CD4 309 [10%], VL <20), Hx IVDU	Teevir (TDF + FTC + EFV)	No	14 300 000	6	TN	No
4	SOF/VEL	12	42	M	Yes (CD4 727 [23%], VL <20), MSM	3TC + DRV + RTV	No	16 500 000	—	TN	No
5	SOF/VEL	12	39	F	Yes (CD4 781 [44%]), multiple partner	Teno‐EM + RPV	No	2 540 000	3	TN	Yes

3TC, lamivudine; ART, anti‐retroviral; DAC, daclatasvir; DRV, darunavir; EFV, efavirence; F, female; FTC, emtricitabine; GT, genotype; HCV, hepatitis C virus; HIV, human immunodeficiency virus; Hx IVDU, history of intravenous drug user; LDV, ledipasvir; M, male; MSM, hen have sex with men; NVP, nevirapine; RPV, rilpivirine; RTV, ritonavir; SOF, sofosbuvir; TDF, tenofovir; TE, treatment experience; Teno‐EM, tenofovir and emtricitabine; TN, treatment naïve; VEL, velpatasvir; VL, viral load.

## Discussion

We retrospectively examined the treatment outcomes for HCV in patients with and without HIV/HCV co‐infection. The overall SVR rate (96.9%) was comparable to previously reported findings.^15^, ^16^, ^17^, ^18^ The rate of SVR in real‐world setting was not different between the HCV mono‐infection group and the HIV/HCV co‐infection group (96.9% *vs* 96.9%, respectively). Several previously reported studies have shown the same response.^19^, ^20^, ^21^, ^22^, ^23^ In contrast to our findings, Gayam *et al*. reported lower rate of SVR in HIV/HCV co‐infection group (84% *vs* 94%).^24^ In this study, the majority of patients was no/mild liver fibrosis by APRI score (68%) and GT1 was predominant genotype (66%), as well as the HIV status was good regarding high CD4 count and low HIV viral load, which may explain the high SVR rate.

In addition, patients with GT6 HCV infection had lower rate of SVR in our study. Our finding was the same as previously reported real‐word setting outcomes in Thailand.^16^ This could potentially be explained by limited efficacy of SOF‐based regimens for GT6 infection and the small sample size. In contrast, Two studies from New Zealand (*n* = 25) and United States (*n* = 64) reported overall SVR rate was 96% and 95% respectively with 12 weeks of SOF/LDV.^25^, ^26^ Another study from Myanmar reported lower SVR rate of 64% in patients with GT6 infection without cirrhosis and 42% in patients with cirrhosis using SOF/LDV combination.^27^

Based on the univariate analysis, patients treated with SOF/VEL regimen were statistically significantly less likely to achieve SVR (*P*‐value 0.011). However, due to the small sample size of patients receiving SOF/VEL in our study (*n* = 10), the result should be cautiously interpreted as the difference may not be as profound with a larger sample size. In the group of SOF/VEL regimen, all patients in GT6 (two patients) achieved SVR, and two patients who did not achieve SVR were GT3 and unknown GT. Seven patients were in HIV/HCV co‐infection group and ART was changed from EFV or NVP to rilpivirine or DTG and no history of using PPI(proton pump inhibitor) during treatment of hepatitis C. Because this study was done before the new government policy, SOF/VEL as pangenotypic regimen, so the number of SOF/VEL groups was quite low.

Interestingly, more than half of the patients in our cohort who did not achieve SVR had GT6 diseases. This could potentially be explained by the treatment regimens utilized (SOF/LDV; *n* = 2, SOF/DAC; *n* = 1). Our findings are different from previously reported 95.3% SVR rate in SOF/LDV treated patients with GT6,^26^ which could potentially be explained by the small number (*n* = 18) of GT6 patients in our cohort. In another study from Thailand, a large cohort SOF‐based study showed high rate of SVR 12 in GT6 (96.7%).^28^ However, the Thai government has recently approved SOF/VEL regimen, which could potentially lead to improved treatment outcomes for patients with GT6 diseases in the future.

Three of five patients who did not achieve SVR 12, already received treatment, SOF/VEL and RBV 12 weeks regimen was provided and treatment results were in the follow‐up stage.

Because the study was conducted before SOF/VEL was supported by the government policy, this study had a small number of patients receiving SOF/VEL. The majority of patients in this study received SOF/LDV, which was supported by the government policy at that time.

Our study has certain limitations. First, the sample size was small, especially for the HCV mono‐infection cohort, the number of patients in some treatment regimens, and the number of patients with specific viral genotypes. This could lower the power to detect the statistical differences and provide meaningful conclusions. Moreover, the retrospective nature of the study did not allow certain clinical data collection, especially patient‐reported outcomes. Finally, the viral resistant testing was not available. Nonetheless, we reported the real‐world outcomes of SOF‐based regimens in a large cohort of patients with HIV/HCV co‐infection in Thailand.

In the real‐world setting, sofosbuvir‐based regimen appears to be effective and well tolerated in both HIV/HCV co‐infected and HCV mono‐infected patients. Our findings should be confirmed in a larger study with viral resistant testing to provide insight into cases with poor outcomes.
